# Causal Association Between Inflammatory Bowel Disease and Psoriasis: A Two-Sample Bidirectional Mendelian Randomization Study

**DOI:** 10.3389/fimmu.2022.916645

**Published:** 2022-06-10

**Authors:** Yajia Li, Jia Guo, Ziqin Cao, Jianhuang Wu

**Affiliations:** ^1^ Department of Dermatology, Xiangya Hospital, Central South University, Changsha, China; ^2^ National Clinical Research Center for Geriatric Disorders, Xiangya Hospital, Central South University, Changsha, China; ^3^ Department of Spine Surgery and Orthopaedics, Xiangya Hospital, Central South University, Changsha, China

**Keywords:** inflammatory bowel disease, Crohn’s disease, ulcerative colitis, psoriasis, psoriatic arthritis, Mendelian randomization

## Abstract

**Background:**

Previous observational studies have found an association between inflammatory bowel disease (IBD) and psoriasis. Using the mendelian randomization (MR) approach, we aim to determine whether there was a causal association between IBD and psoriasis.

**Methods:**

We performed a two-sample MR with the genetic instruments identified for IBD and its main subtypes, Crohn’s disease (CD) and ulcerative colitis (UC), from a genome-wide association study (GWAS) involving 25,042 cases with an IBD diagnosis and 34,915 controls. Summarized data for psoriasis were obtained from different GWAS studies which included 4510 cases and 212,242 controls without psoriasis. Causal estimates are presented as odds ratios (ORs) with 95% confidence intervals (CIs).

**Results:**

The overall outcome of MR analysis was to demonstrate that genetic predisposition to IBD was associated with an increased risk of psoriasis (OR: 1.1271; 95% CI: 1.0708 to 1.1864). Psoriatic arthritis (PsA) had a significant association with total IBD (OR: 1.1202; 95% CI: 1.0491 to 1.1961). Casual relationship was also identified for CD-psoriasis (OR: 1.1552; 95% CI: 1.0955 to 1.2182) and CD-PsA (OR: 1.1407; 95% CI: 1.0535 to 1.2350). The bidirectional analysis did not demonstrate that a genetic predisposition to psoriasis was associated with total IBD, although psoriasis showed association with CD (OR: 1.2224; 95% CI: 1.1710 to 1.2760) but not with UC. A genetic predisposition to PsA had a borderline association with IBD (OR: 1.0716; 95% CI: 1.0292 to 1.1157) and a suggestive association with CD (OR: 1.0667; 95% CI: 1.0194 to 1.1162).

**Conclusion:**

There appears to be a causal relationship between IBD and psoriasis, especially for PsA, but for psoriasis and IBD, only total psoriasis and PsA were associated with CD. Understanding that specific types of psoriasis and IBD constitute mutual risk factors facilitates the clinical management of two diseases.

## Introduction

Psoriasis is an immune-related, chronic inflammatory skin disorder of common occurrence. Clearly demarcated areas of erythematous plaques with overlying silvery scales appear on the skin. There are estimated to be 60 million psoriasis patients worldwide with the prevalence ranging from 0.1% to 1.99%. The conditions occur more frequently in high-income countries and approximately 2% of the population in Western and Central Europe are affected (1, 2). Psoriasis vulgaris is the most common form, accounting for 90% of cases (3). Psoriatic arthritis (PsA), involving inflammation, swelling, and pain in affected joints, may precede or succeed skin manifestations, and PsA affects 5% to 30% of psoriasis patients (4–6). Psoriatic disease, consisting of both psoriasis (only skin manifestation) and PsA, is considered a multifactorial disease that has been associated with multiple systemic disorders. Numerous studies involving diverse populations and settings (including both the western and eastern countries) have associated psoriatic diseases with autoimmune, metabolic, gastrointestinal, and mental health disorders (4, 5, 7–10), which has caused a worldwide disease burden. 

Inflammatory bowel disease (IBD), including Crohn’s disease (CD) and ulcerative colitis (UC), involves chronic relapsing episodes of immune-mediated inflammation. Evidence from meta-analyses and observational studies supports an underlying bidirectional relationship between psoriasis and inflammatory bowel disease (IBD), although mechanisms remain unclear (11, 12). Many factors, such as common genetic susceptibility loci, clinical course, and immunologic features, are shared by psoriatic diseases and IBD (5, 13–16), and IBD was considered a subsequent extra-articular manifestation of PsA which should be specially monitored in PsA patients according to the previous studies. However, basic and observational studies are susceptible to confounding factors, such as demographics or environmental exposure (17, 18). Thus, any causal bidirectional role in the respective development of psoriasis/PsA and IBD remains controversial.

Causal relationships between exposure and outcome can be assessed by Mendelian randomization (MR) analysis introducing genetic variation as an instrumental variable. This approach exploits the random distribution of genetic variation to eliminate confounding factors and reverse causality, simulating the randomization process of a randomized controlled experiment. The current study applied MR to examine the causal relationship, the strength of association, and the direction of causality between psoriasis and IBD (19–21).

## Materials and Methods

**Figure 1:**
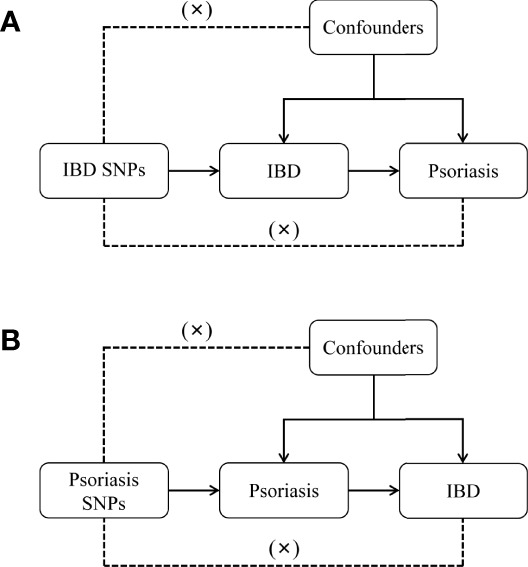
Diagram for key assumptions of MR analyses. **(A)** IBD SNPs were used as the genetic instruments to investigate the causal effect of IBD on psoriasis. **(B)** Psoriasis SNPs were used as the genetic instruments to investigate the causal effect of psoriasis upon IBD. Line with arrows indicates that the genetic instruments (SNPs) are associated with the exposure and could only affect the outcome *via* the exposure. Dashed lines indicate that the genetic instruments (SNPs) are independent of any confounding variables between the results. IBD, inflammatory bowel disease.

Multiple single-nucleotide polymorphisms (SNP) representing genetic variation were selected as instrumental variables and subjected to two-sample MR analysis. Three key hypotheses were adopted as follows (**Figure 1**): 1. instrumental variables are directly related to exposure; 2. instrumental variables are independent of any confounding variables; 3. genetic variants only affected results *via* exposure (22). MR analysis was used to assess bidirectional causal relationships between psoriasis and IBD, including subtypes of psoriasis, psoriasis vulgaris, and PsA, and of IBD, UC and CD.

### Genome-Wide Association Analysis

Databases resulting from genome-wide association studies (GWAS), including GWAS Catalog, IEU openGWAS and NealELab, were searched and eligible datasets extracted. Since all data used was already in the public domain, no additional ethical approval was required. The study population’s genetic background was restricted to that of European ancestry to reduce the resulting bias caused by ethnically related confounding factors. The FinnGen Biobank Analysis Consortium 2021 (23) was used to identify genetic risk variants for psoriasis. The database included 4510 cases with a psoriasis diagnosis plus a control group of 212,242 individuals without psoriasis. A dataset identifying 334 cases of psoriasis vulgaris and 1,637 of PsA in addition to 212,242 controls was used for subtype analysis. The diagnosis of psoriasis and its subtypes was according to the ICD-10 (International Classification of diseases) criteria.

Similarly, the European Bioinformatics Institute (EBI) database included 25,042 cases with an IBD diagnosis and 34,915 controls. Datasets identifying 12,366 cases of UC with 33,609 controls and 12,194 cases of CD with 28,072 controls were used to assess secondary outcomes. The diagnosis of IBD and its subtypes was based on accepted endoscopic, histopathological, and radio­logical criteria.

### SNP Selection

A genome-wide significance level of p < 5 · 10^-8^ and a clumping algorithm with a cutoff of r^2^ = 0.001 and kb = 10000 were used to avoid linkage disequilibrium (LD). For exposure analyses of IBD and its two main subtypes, 95 SNPs related to total IBD, 76 SNPs related to CD, and 51 SNPs related to UC were selected. There were an additional 10 SNPs associated with total psoriasis selected for co-analysis with IBD. At a threshold of p < 5 x 10^-8^, fewer than 10 SNPs were identified for the two subtypes of psoriasis thus failing to meet the minimum requirements for MR studies (24, 25). At a looser threshold of *p* < 5 · 10^-6^ (26, 27), 10 SNPs for psoriasis vulgaris and 13 SNPs for PsA were identified. Due to the reduced significance threshold, *F* statistics were used to evaluate the risk of weak instrument bias, producing a bias level of *F <*10. Selected SNPs were also matched to databases for phenome-wide association studies (pheWAS) to avoid the potential association between the SNPs and outcomes confounders with a threshold of *p* < 5 x 10^-6^ (26, 28).

Exposures and outcomes were harmonized in terms of effect allele and subsequent analyses based on the merged exposure-outcome dataset. Detailed information on IVs is shown in **Supplementary Tables 1–6**.

### Statistical Analysis

A meta-analytical approach involving an inverse variance weighted model (IVW) was used to combine Wald estimates of causality for each IV, an approach expected to be stable with balanced pleiotropy. Several statistical approaches were combined to evaluate associations between cause and effect. Weighted median estimator model, weighted model-based method, MR-Egger regression model, MR-Robust Adjusted Profile Score (MRAPS), and MR pleiotropy residual sum and outlier (MR-PRESSO) were also established to estimate causal associations under different conditions. The weighted median estimator method examined the median effects of all available SNPs when half the IVs were valid, resulting in unbiased estimates of effects (29). The weighted model-based method obtained robust overall causal estimates when the majority of individual estimates were from valid IVs (30). The MR-Egger regression model provided a relatively robust estimate independent of IV validity and an adjusted result by existing horizontal pleiotropy *via* the regression slope and intercept (31, 32). MRAPS has the potential to derive a more accurate causal assessment on the condition of ideal independence of IVs (33). MR-PRESSO used global and SNP-specific observed residual sum of squares to test for potential outlying IVs to obtain a corrected causal effect by excluding outliers with *p* < 0.05 in the further distortion test (34). Heterogeneity of IVs was assessed by Cochrane’s Q-statistic. A value of *p* < 0.05 indicated significant heterogeneity in which case a random-effect model was adopted for subsequent analyses. Otherwise, a fixed-effect model was used (35). IVs were excluded one by one to judge the stability of the MR results (36). Directional pleiotropy was assessed and corrections were made based on the intercept obtained from the MR-Egger regression model analysis (32). However, compared to the IVW method, other methods produced wide confidence intervals (CI) (37) and were only used as complementary methods. Thus, the MR-Egger Regression Model was used in the event of significant pleiotropy and the MR-PRESSO model for the detection of final outliers. Otherwise, IVW results were given priority.

Causal estimates are presented as odds ratios (ORs) with 95% confidence intervals (CIs). A Bonferroni correction was made to avoid false-positive results of various comparison methods in bidirectional multiple tests. A two-sided p-value <0.0083 was regarded as statistically significant after Bonferroni correction while associations with p-values ≥0.0083 and <0.05 were regarded as suggesting significance. Bidirectional two-sample MR analysis was conducted using open-source statistical software R (version 4.1.2, R Foundation for Statistical Computing, Vienna, Austria) with TwoSampleMR (version 0.5.6) and MR-PRESSO packages (version 1.0.0). Results are reported following the STROBE-MR (Strengthening the Reporting of Observational Studies in Epidemiology—Mendelian Randomization) statement (38).

## Results

### Primary MR Analysis of Bidirectional Associations Between IBD and Psoriasis

Significant heterogeneity was found by Cochran’s Q test (Q=206.263; *p*= .001) and an IVW approach with the multiplicative random-effect model was applied to the main analyses. No directional pleiotropy was found by MR-Egger regression analysis (intercept = 0.0081; se = 0.0218; p = 0.2357). After the removal of 3 distorted outliers (rs938650, rs2301127, rs7608697), genetically predicted IBD was positively associated with psoriasis (IVW: OR:1.1268; 95% CI: 1.0662 to 1.1908; *p*=2.35E-5). The association was supported by MR-PRESSO analysis (outlier-corrected: OR: 1.1271; 95% CI: 1.0708 to 1.1864; *p*=1.50E-05).

No directional pleiotropy was shown in MR-Egger regression (intercept = 0.0250; se = 0.0218; p = 0.3835) for the influence of psoriasis on IBD. Significant heterogeneity was found by Cochran’s Q test (Q=79.86832; *p*=.001) and three outliers (rs13210419, rs10829130, rs9481169) were identified. The results did not indicate a directly causal effect of psoriasis on IBD but significance was suggested (IVW: OR: 1.1614; 95% CI: 1.0357 to 1.3022; *p*=1.04E-02). After removal of outliers, the association was not significant (outlier-corrected: OR: 1.1027; 95% CI: 0.9624 to 1.2634; *p*=2.54E-01).

**Figure 2:**
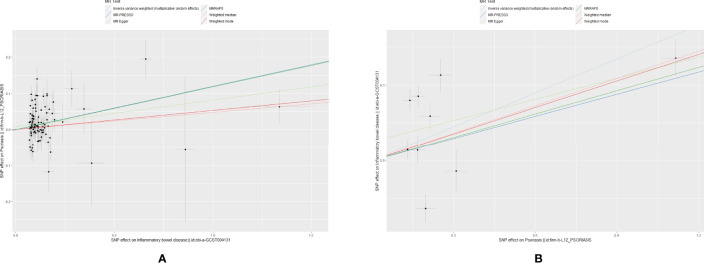
Scatter plots of primary MR analysis. The slope of each line corresponding to the estimated MR effect in different models. **(A)** IBD on psoriasis; **(B)** Psoriasis on IBD.

**Figure 3:**
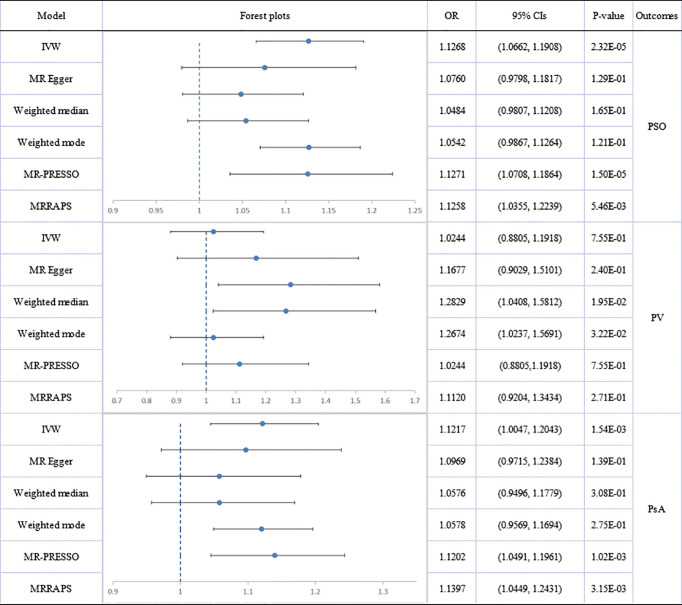
Causal estimates given as odds ratios (ORs) and 95% confidence intervals for the effect of inflammatory bowel disease on psoriasis as a whole, psoriasis vulgaris and psoriatic arthritis. IBD, inflammatory bowel disease; PSO, psoriasis; PV, psoriasis vulgaris; PsA, psoriatic arthritis.

**Figure 4:**
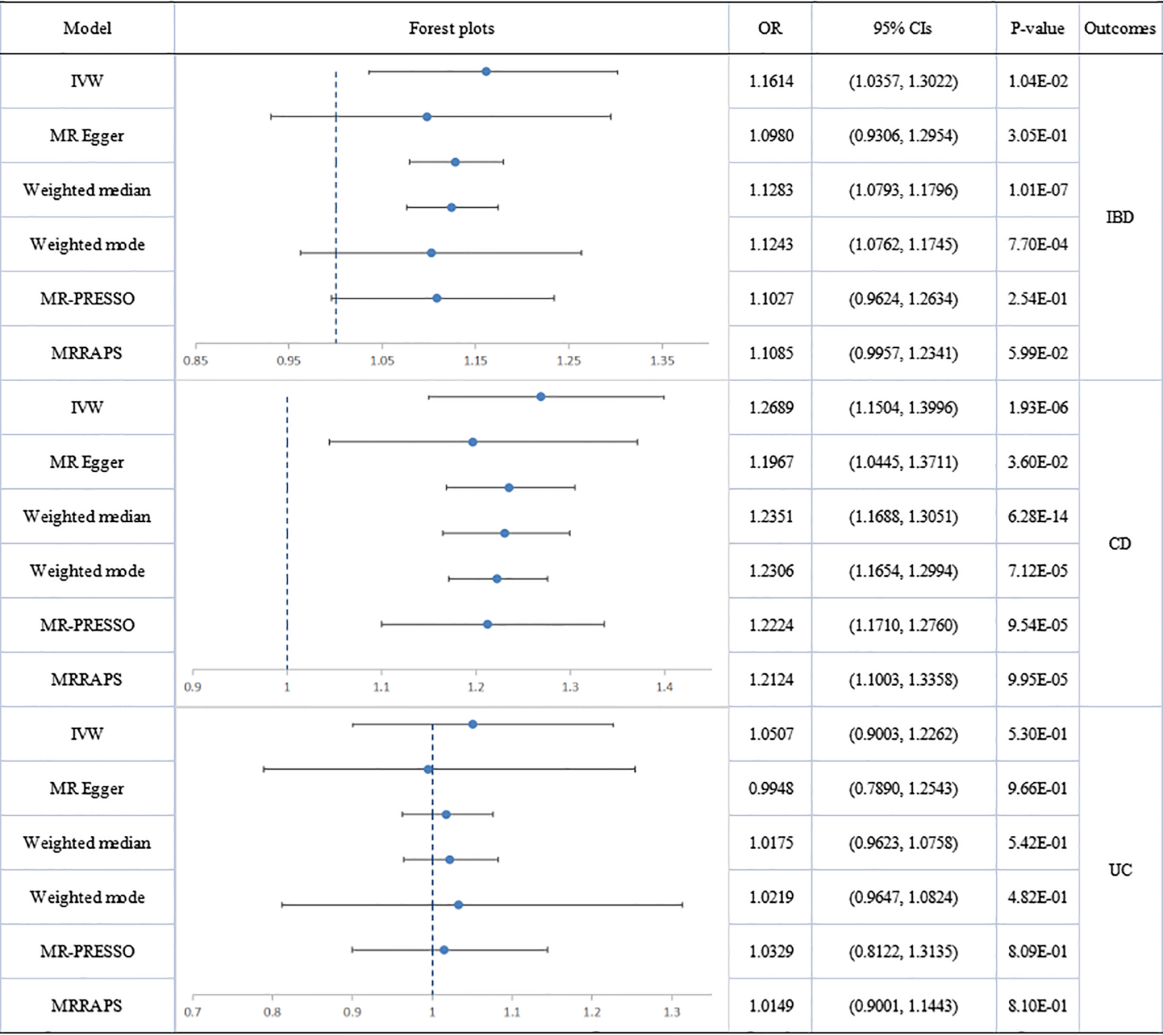
Causal estimates given as odds ratios (ORs) and 95% confidence intervals for the effect of psoriasis on inflammatory bowel disease, Crohn’s disease and ulcerative colitis. IBD, inflammatory bowel disease; CD, Crohn’s disease; UC, ulcerative colitis; PSO, psoriasis.

The scatter plots of causal relationships of primary MR analysis are shown in **Figure 2**. The forest plots of causal relationships of genetically predicted IBD and the risk of psoriasis and its subtypes are shown in **Figure 3**, and the forest plots of causal effects of psoriasis on IBD and its subtypes are shown in **Figure 4**.

### Secondary MR Analysis

#### Effect of IBD or Its Main Subtypes on Psoriatic Diseases

Analyses of total IBD on psoriasis vulgaris or PsA showed significant heterogeneity but no directional pleiotropy. Hence, multiplicative random-effect models were applied in the IVW analysis and a significant association emerged between total IBD and PsA (IVW: OR: 1.1217; 95% CI: 1.0047 to 1.2043; p=1.54E-03). There was no association with psoriasis vulgaris. MR-PRESSO confirmed the IBD-PsA association (outlier-corrected: OR: 1.1202; 95% CI: 1.0491 to 1.1961; *p*=1.02E-03).

Analyses of IBD’s two main subtypes, UC and CD, were also conducted. The association of CD with psoriatic diseases showed significant heterogeneity but no directional pleiotropy. CD was causally associated with total psoriasis (IVW: OR: 1.1573; 95% CI: 1.0728 to 1.2485; p=1.58E-04) and with PsA (IVW: OR: 1.1431; 95% CI: 1.0415 to 1.2545; p=4.84E-03) but not with psoriasis vulgaris. Results of MR-PRESSO analysis confirmed these findings, showing a significant CD-psoriasis (outlier-corrected: OR: 1.1552; 95% CI: 1.0955 to 1.2182; p=1.13E-06) and CD-PsA association (outlier-corrected: OR: 1.1407; 95% CI: 1.0535 to 1.2350; p=1.77E-03).

However, no causal association was found between UC and total psoriasis or the two subtypes.

#### Effect of Psoriatic Diseases on IBD and Its Main Subtypes

The association of total psoriasis with CD and UC showed heterogeneity but no directional pleiotropy and multiplicative random-effect models were applied for the final IVW analysis. Psoriasis was significantly associated with CD (IVW: OR: 1.2689; 95% CI: 1.1504 to 1.3996; p=1.93E-06). Two outliers (rs12713428 and rs2021511) were removed and the association remained significant (outlier-corrected: OR: 1.2224; 95% CI: 1.1710 to 1.2760; p=9.54E-05). However, there was no evidence of a causal effect of psoriasis on UC.

Significant heterogeneity but no directional pleiotropy was found in the MR analyses of the relationships between PsA and IBD plus subtypes. The IVW model did not show a causal effect of PsA on IBD and its subtypes. However, after removing all outliers, the MR-PRESSO model did reveal a causal effect for PsA on IBD (outlier-corrected: OR: 1.0716; 95% CI: 1.0292 to 1.1157; p=7.28E-03) and suggested a significant causal effect of PsA on CD (outlier-corrected: OR: 1.0667; 95% CI: 1.0194 to 1.1162; p=1.91E-02). No heterogeneity, no directional pleiotropy, and no significant causal association were found between psoriasis vulgaris and IBD plus subtypes.

The forest plots and scatter plots of secondary MR analysis are shown in **Supplementary Figures 1–4**. Details of sensitivity analysis are shown in **Table 1** and **Supplementary Figures 5, 6**.

**Table 1:** Sensitivity analyses of MR.

Exposure	Outcome	Number of IVs	Heterogeneity test		MR-Egger pleiotropy test		MR-PRESSO global outlier test		
			Q	P-value	Intercept	P-value	RSSobs	P-value	Outlier
IBD	PSO	92	209.4230	0.0000	0.0081	0.2357	217.0734	<1E-04	rs938650, rs2301127, rs7608697
	PV	92	131.2260	0.0068	-0.0228	0.2209	136.8737	4.80E-03	rs113846785, rs11548656, rs755374
	PSA	93	134.8886	0.0037	0.0039	0.6569	138.0988	5.00E-03	rs11669299, rs17656349
CD	PSO	72	331.6052	0.0000	0.0122	0.4775	338.1244	<2E-04	rs111281598, rs2021511, rs755374, rs9501641
	PV	74	142.5384	0.0000	-0.0026	0.9477	145.2784	<2E-04	rs111281598, rs755374
	PSA	74	195.1236	0.0000	0.0380	0.0689	200.3732	<2E-04	rs111281598, rs9501641
UC	PSO	43	270.2515	0.0000	0.0389	0.1238	290.6522	<2E-04	rs10884966, rs11683692, rs1583792, rs34004493, rs4316387, rs4807570, rs56116661, rs5754100
	PV	50	70.3035	0.0307	-0.0073	0.8702	73.7106	2.96E-02	rs13200059
	PSA	48	150.8000	0.0000	0.0321	0.2912	159.4384	<2E-04	rs28383224, rs9260809, rs9271176
PSO	IBD	6	79.8683	0.0000	0.0250	0.3835	102.6720	<2E-04	rs13210419, rs10829130, rs9481169
	CN	7	37.3362	0.0000	0.0270	0.2740	48.0208	4.76E-02	rs12713428, rs2021511
	UC	6	84.4808	0.0000	0.0236	0.5420	103.0488	4.00E-04	rs13210419, rs10829130, rs9481169
PV	IBD	9	8.6056	0.3767	0.0019	0.9352	12.1724	3.45E-01	None
	CN	9	6.4970	0.5917	0.0274	0.3281	9.7219	5.31E-01	None
	UC	9	12.29409 8	0.1386	-0.0208	0.5488	15.5161	1.70E-01	None
PsA	IBD	11	69.7408	0.0000	-0.0069	0.7814	80.6961	<2E-04	rs13033143, rs696
	CN	11	52.1325	0.0000	0.0049	0.8602	59.7108	<2E-04	rs13033143, rs696
	UC	11	65.6526	0.0000	-0.0275	0.3641	80.1913	<2E-04	rs2523560, rs696

MR, Mendelian randomization analysis; nIVs, Number of instrumental variables. IBD, inflammatory bowel disease; CD, Crohn’s disease; UC, ulcerative colitis; PSO, psoriasis; PV, psoriasis vulgaris; PsA, psoriatic arthritis.

## Discussion

The overall outcome of MR analysis was to demonstrate that genetic predisposition to IBD was associated with an increased risk of psoriasis (OR:1.1268; 95% CI: 1.0662 to 1.1908). Total IBD had a significant association with PsA (OR: 1.1217; 95% CI: 1.0047 to 1.2043) but no association was found for psoriasis vulgaris. For the subtypes of IBD, a relationship was suggested for CD and PsA (OR: 1.1431; 95% CI: 1.0415 to 1.2545) while none was present for UC. Neither subtype of IBD was associated with psoriasis vulgaris. However, the bidirectional analysis did not demonstrate that a genetic predisposition to psoriasis was associated with total IBD (OR: 1.1027; 95% CI: 0.9624 to 1.2634), although psoriasis was associated with CD (OR: 1.2689; 95% CI: 1.1504 to 1.3996) but not with UC. A genetic predisposition to PsA had a borderline association with IBD (OR: 1.0716; 95% CI: 1.0292 to 1.1157) and a suggestive association with CD (OR: 1.0667; 95% CI: 1.0194 to 1.1162). Psoriasis vulgaris was not genetically associated with either CD or UC.

There has been much discussion in the medical literature, including two meta-analyses, on the relationship between psoriasis and IBD. A meta-analysis of 9 observational studies (5 case-control or cross-sectional and 4 cohorts) (11), including 7,794,087 study participants, concluded that patients with psoriasis had increased odds of CD (OR: 1.70; 95% CI: 1.20 to 2.40) and UC (OR: 1.75; 95% CI: 1.49 to 2.05), as well as an increased risk ratio (RR) of CD (RR: 2.53; 95% CI: 1.65 to 3.89) and UC (RR 1.71; 95% CI, 1.55;1.89). A pooled sub-analysis of the 2 cohort studies demonstrated a significantly increased risk of CD (RR: 2.74; 95% CI: 1.41 to 5.32) and a non-significantly increased risk of UC in patients with PsA. Furthermore, Alinaghi et al. (12) analyzed 93 studies and reported bidirectional associations between psoriasis and IBD. IBD was associated with a 1.8-fold increased odds for psoriasis and associations between psoriasis and CD (OR: 2.0; 95% CI: 1.4 to2.9) and UC (OR: 1.5; 95% CI: 1.2 to2.0) were also observed. Similarly, patients with psoriasis also had increased odds of developing total IBD (OR: 2.0; 95% CI: 1.6 to 2.5), CD (OR: 2.2; 95% CI: 1.6 to 3.1) and UC (OR: 1.6, 95% CI: 1.3 to 2.0). There are some discrepancies between these reported estimates and our results. We believe that the difference may be caused by the two different analysis methods themselves. The observational research might be affected by unavoidable clinical confounding factors, these confounding factors could affect both exposure and outcome and weaken the ability of observational results to make an accurate causal judgment. Therefore, even if an observational study reported a strong correlation, it could not prove the existence of a direct causal correlation. Mendelian randomization could avoid the influence of these confounding factors by introducing genetic instrumental variables, to obtain a relatively accurate causal assessment.

The results of the current work demonstrate that the observational estimates reported in the literature can be explained by a causal effect of IBD on psoriasis. Thus, the presence of IBD was partly responsible for the development of psoriasis in some patients with the relationship being especially strong for PsA. It is noteworthy that, when the exposure was psoriasis, there was some evidence of causality in the case of CD but not in that of UC. For both psoriasis and IBD, disease subtypes differed in terms of their bidirectional associations. UC was not genetically linked to any subtype of psoriasis and psoriasis vulgaris was not linked to any type of IBD. Subtypes of the same broad group of diseases may share some similarities in the genetic background but there remain significant differences in individual genes, genetics, and immunity between PsA and psoriasis vulgaris (1, 39). The same may be said for UC and CD (40–42). Further study is required to elucidate whether such apparent differences have their origins in environmental factors, differences in immune response, changes in intestinal flora or genetic factors that influence different disease subtypes.

We would like to highlight some strengths of our study and acknowledge some limitations. Firstly, it is the first study to assess the bidirectional causal association of IBD and psoriasis by using a 2-sample MR approach. This method is less susceptible to confounders, reverse causation, and exposures non-differentially compared to observational studies. Secondly, the subtypes of the disease are strictly defined to avoid the impact on the results of the co-existence of diseases. Thirdly, sensitivity analysis was performed to ensure the consistency of causal estimation and the robustness of the results. There are also some limitations. Due to the paucity of IVs, a lower p threshold was chosen for the study of IBD with PsA and psoriasis vulgaris. Although no significant risk of weak instrument bias was found in our F-statistic tests, this negative result should be interpreted with caution. In addition, the sample size of psoriasis vulgaris patients was relatively small due to the strict definition. The current study was conducted on individuals of European ancestry and cannot be reliably generalized to other races. For example, some gastrointestinal involvements (such as IBD) were also identified as extra-articular manifestations of Chinese patients with PsA (9). Although previous studies revealed that the prevalence of PsA among Asian psoriasis patients might be less than that of western patients (43, 44), the exploration of the causal association between psoriatic diseases and IBD in the Asian population in the future studies remained to be of great significance. Last but not the least, although we have matched all selected SNPs to the pheWAS database to avoid the potential confounders and associated horizontal pleiotropy with a strict threshold, this measure can not completely avoid the impact of horizontal pleiotropy as the exact biological function of many genetic variants is still unknown. Future studies have been planned to address these limitations when higher quality GWAS studies become available.

## Conclusions

In conclusion, there appears to be a causal impact of IBD on psoriasis, especially for PsA. Notable differences between the two subtypes of IBD, UC, and CD, occurred in their respective associations with psoriasis. Total psoriasis and PsA were associated with CD. Understanding that specific types of psoriasis and IBD constitute mutual risk factors facilitates early diagnosis enabling more efficient targeting of therapy. Further study is required to elucidate the pathophysiology of the causal relationship between IBD and psoriasis.

## Data Availability Statement

The original contributions presented in the study are included in the article/**Supplementary Material**. Further inquiries can be directed to the corresponding authors.

## Author Contributions

ZC and JW conceived the study, participated in its design and coordination, and critically revised the manuscript. YL and ZC searched the databases. ZC, JG, and YL reviewed the GWAS datasets and finished the data collection. ZC and YL finished the data analysis. YL drafted the manuscript. YL, JW, and ZC had full access to all the data collection, analysis, and interpretation. All authors read and approved the final manuscript.

## Funding

This work was supported by the National Natural Sciences Foundation of China (No. 81472073), and the Science Foundation of Hunan Province (No. 2019JJ40518), and theFundamental Research Funds for the Central Universities of Central South University (2022ZZTS0824). The study funders/sponsors had no role in the design and conduct of the study;collection, management, analysis, and interpretation of the data;preparation, review, or approval of the manuscript; and decisionto submit the manuscript for publication.

## Conflict of Interest

The authors declare that the research was conducted in the absence of any commercial or financial relationships that could be construed as a potential conflict of interest.

## Publisher’s Note

All claims expressed in this article are solely those of the authors and do not necessarily represent those of their affiliated organizations, or those of the publisher, the editors and the reviewers. Any product that may be evaluated in this article, or claim that may be made by its manufacturer, is not guaranteed or endorsed by the publisher.
