# Supplementary material for: Causal Association Between Inflammatory Bowel Disease and Psoriasis: A Two-Sample Bidirectional Mendelian Randomization Study
Source: Front Immunol. 2022 Jun 10;13:916645. doi: 10.3389/fimmu.2022.916645 (PMC9226443; doi:10.3389/fimmu.2022.916645)
Supplement: Supplementary file 1 [file DataSheet_1.docx]

**Supplementary Figure 1.** Causal estimates given as odds ratios (ORs) and 95% confidence intervals for the effect of the subtypes of inflammatory bowel disease on psoriasis as a whole, psoriasis vulgaris and psoriatic arthritis. CD: Crohn’s disease; UC: ulcerative colitis; PSO: psoriasis; PV: psoriasis vulgaris; PsA: psoriatic arthritis.

**Supplementary Figure 2.** Causal estimates given as odds ratios (ORs) and 95% confidence intervals for the effect of the subtypes of psoriasis on inflammatory bowel disease, Crohn’s disease and ulcerative colitis. IBD, inflammatory bowel disease; CD: Crohn’s disease; UC: ulcerative colitis; PV: psoriasis vulgaris; PsA: psoriatic arthritis.

**Supplementary Figure 3.** Scatter plots of secondary MR analysis. The slope of each line corresponding to the estimated MR effect in different models. A: IBD on PV; B: IBD on PsA; C: CD on PSO; D: CD on PV; E: CD on PsA; F: UC on PSO; G: UC on PV; H: UC on PsA. IBD, inflammatory bowel disease; CD: Crohn’s disease; UC: ulcerative colitis; PSO: psoriasis; PV: psoriasis vulgaris; PsA: psoriatic arthritis.

**Supplementary Figure 4.** Scatter plots of secondary MR analysis. The slope of each line corresponding to the estimated MR effect in different models. A: PSO on CD; B: PSO on UC; C: PV on IBD; D: PV on CD; E: PV on UC; F: PsA on IBD; G: PsA on CD; H: PsA on UC. IBD, inflammatory bowel disease; CD: Crohn’s disease; UC: ulcerative colitis; PSO: psoriasis; PV: psoriasis vulgaris; PsA: psoriatic arthritis.

**Supplementary Figure 5.** Leave one out sensitivity tests of MR analyses of inflammatory bowel disease and its the subtypes on psoriasis as a whole, psoriasis vulgaris and psoriatic arthritis. Calculate the MR results of the remaining IVs after removing the IVs one by one. A: IBD on PSO; B: IBD on PV; C: IBD on PsA; D: CD on PSO; E: CD on PV; F: CD on PsA; G: UC on PSO; H: UC on PV; I: UC on PsA. IBD, inflammatory bowel disease; CD: Crohn’s disease; UC: ulcerative colitis; PSO: psoriasis; PV: psoriasis vulgaris; PsA: psoriatic arthritis.

**Supplementary Figure 6.** Leave one out sensitivity tests of MR analyses of psoriasis and its the subtypes on inflammatory bowel disease, Crohn’s disease and ulcerative colitis. Calculate the MR results of the remaining IVs after removing the IVs one by one.A: PSO on IBD; B: PSO on CD; C: PSO on UC; D: PV on IBD; E: PV on CD; F: PV on UC; G: PsA on IBD; H: PsA on CD; I: PsA on UC. IBD, inflammatory bowel disease; CD: Crohn’s disease; UC: ulcerative colitis; PSO: psoriasis; PV: psoriasis vulgaris; PsA: psoriatic arthritis.


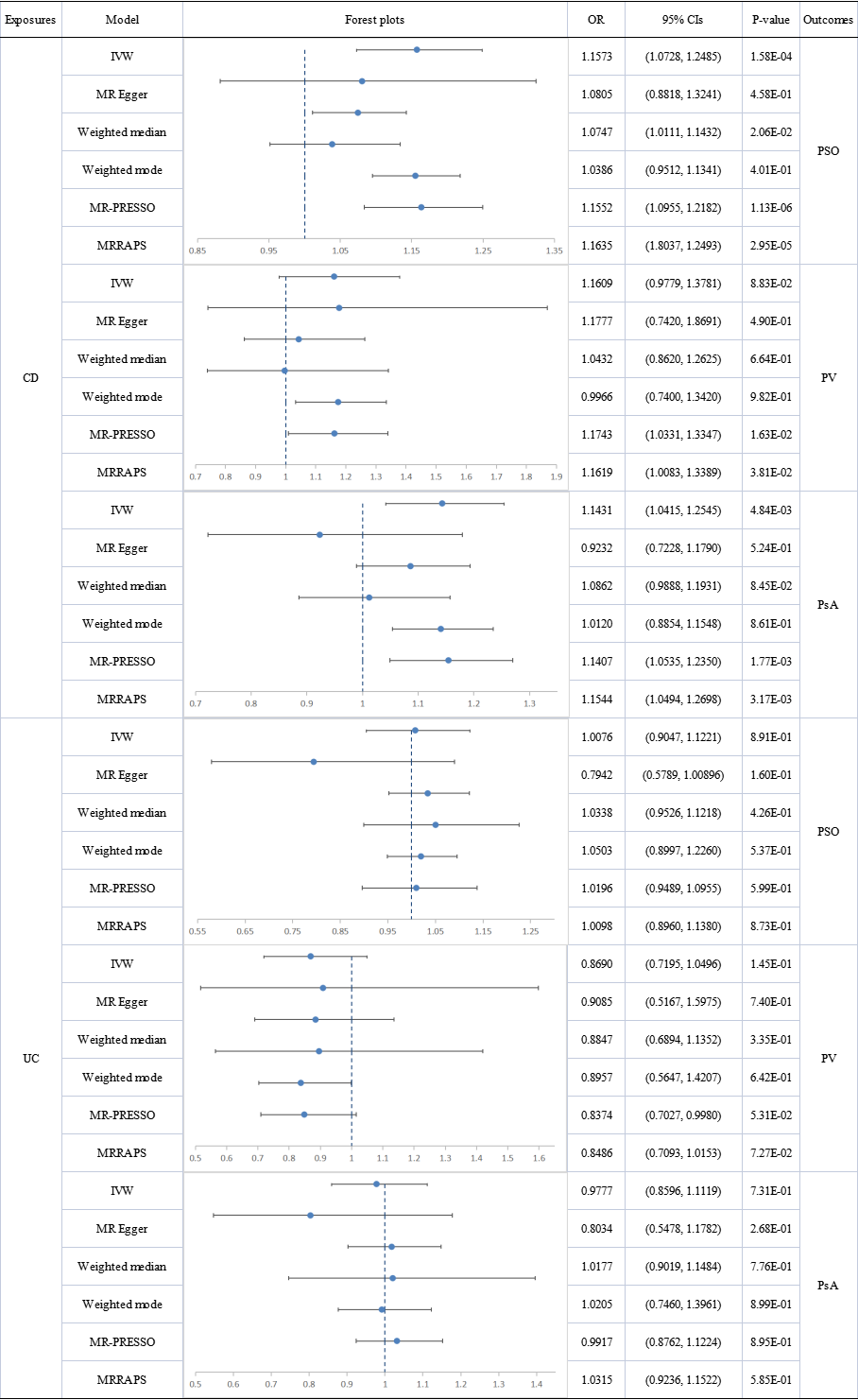


**Supplementary Figure 1.** Causal estimates given as odds ratios (ORs) and 95% confidence intervals for the effect of the subtypes of inflammatory bowel disease on psoriasis as a whole, psoriasis vulgaris and psoriatic arthritis. CD: Crohn’s disease; UC: ulcerative colitis; PSO: psoriasis; PV: psoriasis vulgaris; PsA: psoriatic arthritis.


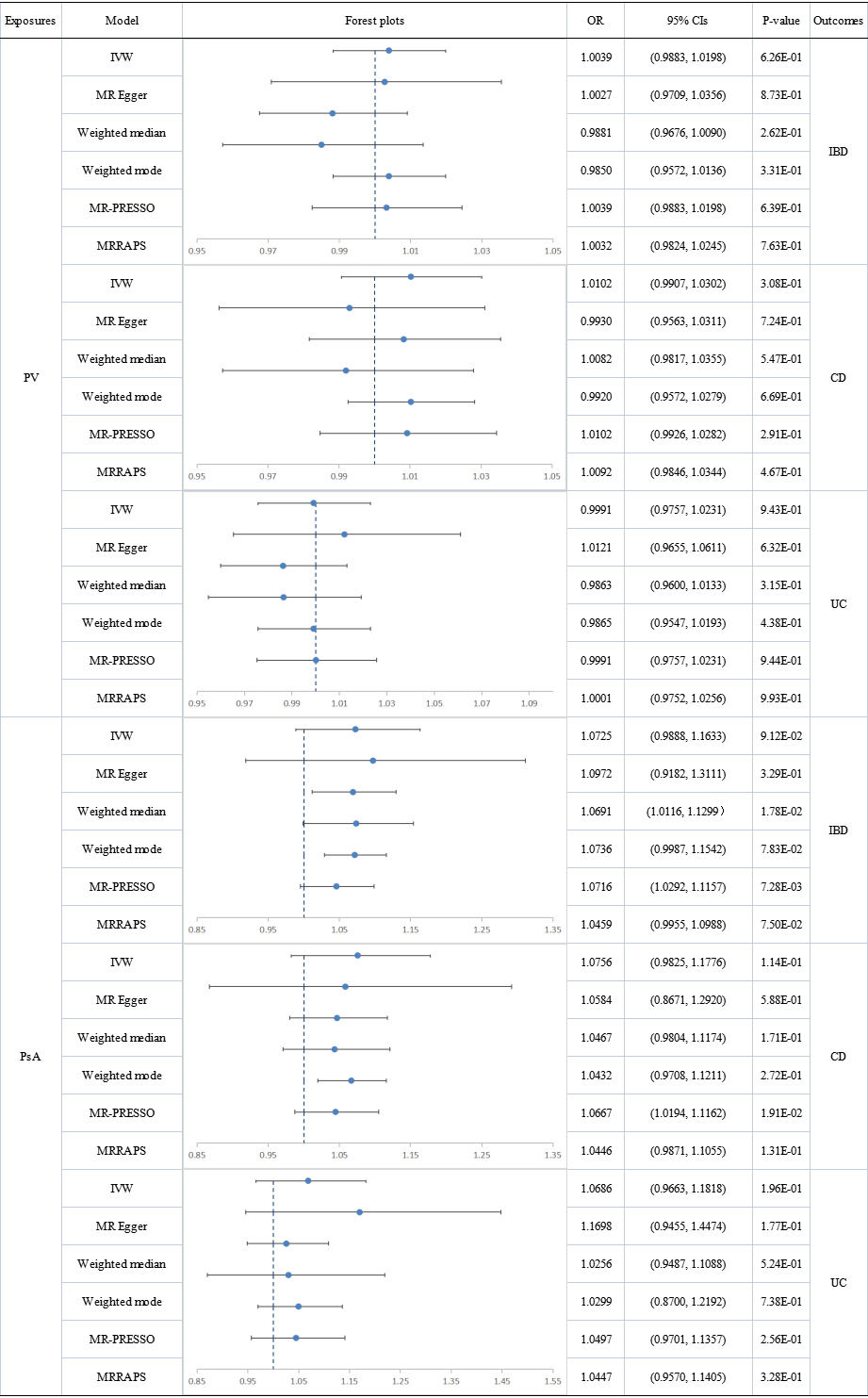


**Supplementary Figure 2.** Causal estimates given as odds ratios (ORs) and 95% confidence intervals for the effect of the subtypes of psoriasis on inflammatory bowel disease, Crohn’s disease and ulcerative colitis. IBD, inflammatory bowel disease; CD: Crohn’s disease; UC: ulcerative colitis; PV: psoriasis vulgaris; PsA: psoriatic arthritis.


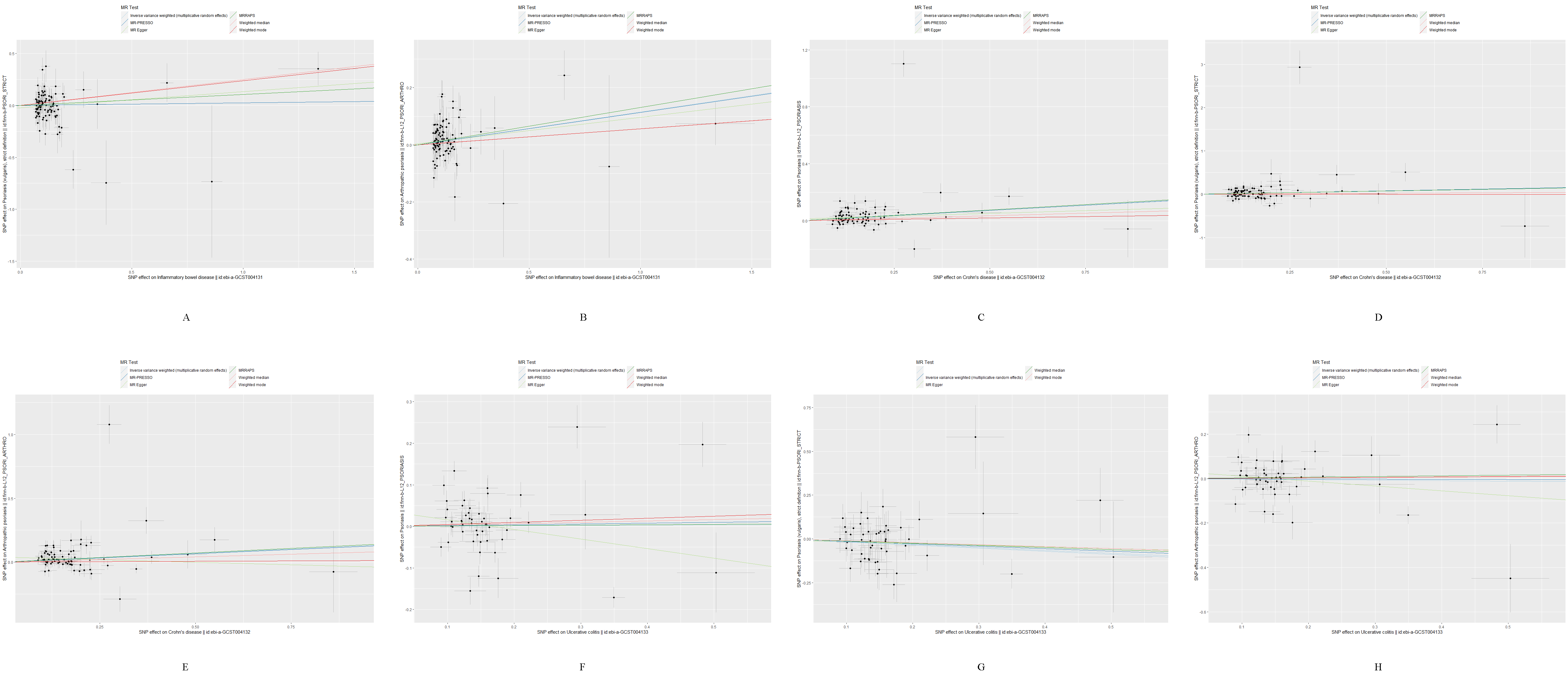


**Supplementary Figure 3.** Scatter plots of secondary MR analysis. The slope of each line corresponding to the estimated MR effect in different models. IBD, inflammatory bowel disease; CD: Crohn’s disease; UC: ulcerative colitis; PSO: psoriasis; PV: psoriasis vulgaris; PsA: psoriatic arthritis. A: IBD on PV; B: IBD on PsA; C: CD on PSO; D: CD on PV; E: CD on PsA; F: UC on PSO; G: UC on PV; H: UC on PsA.


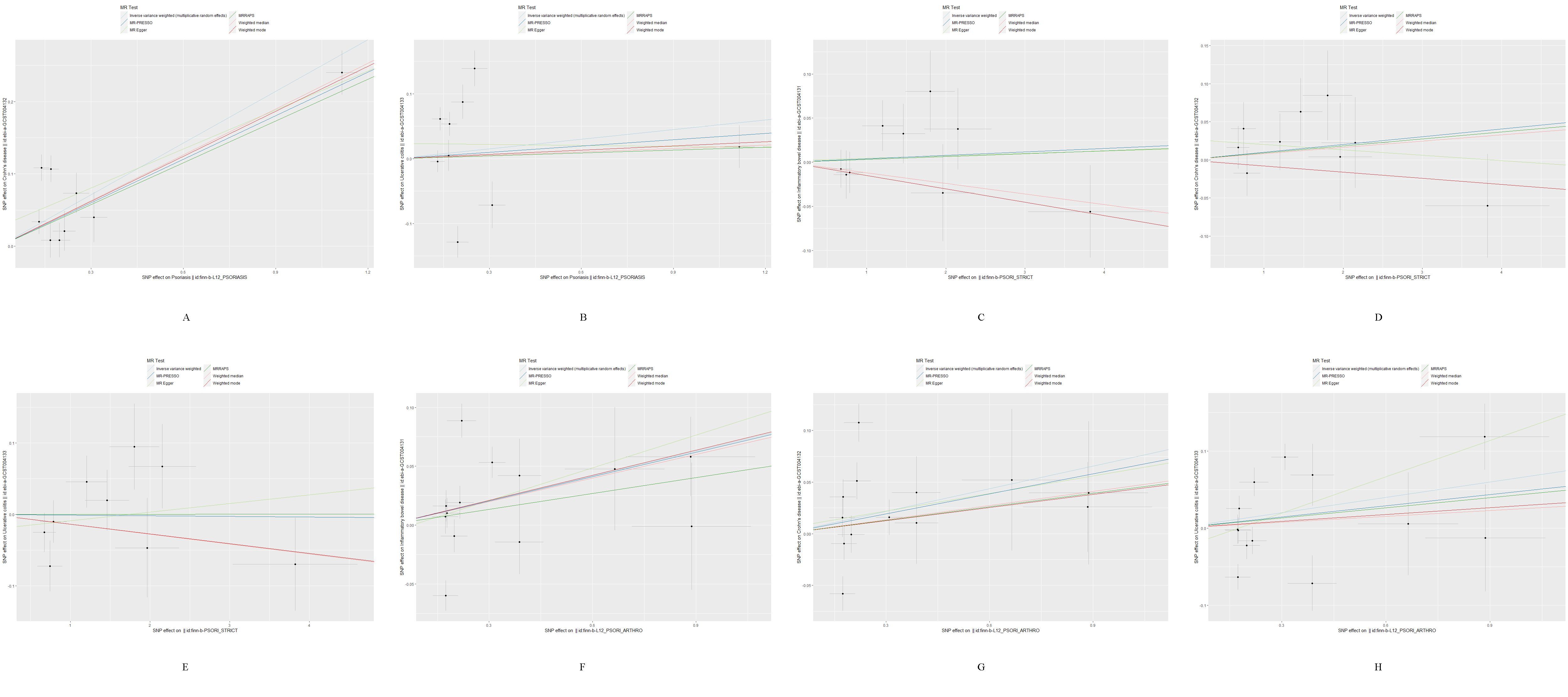


**Supplementary Figure 4.** Scatter plots of secondary MR analysis. The slope of each line corresponding to the estimated MR effect in different models. IBD, inflammatory bowel disease; CD: Crohn’s disease; UC: ulcerative colitis; PSO: psoriasis; PV: psoriasis vulgaris; PsA: psoriatic arthritis. A: PSO on CD; B: PSO on UC; C: PV on IBD; D: PV on CD; E: PV on UC; F: PsA on IBD; G: PsA on CD; H: PsA on UC.


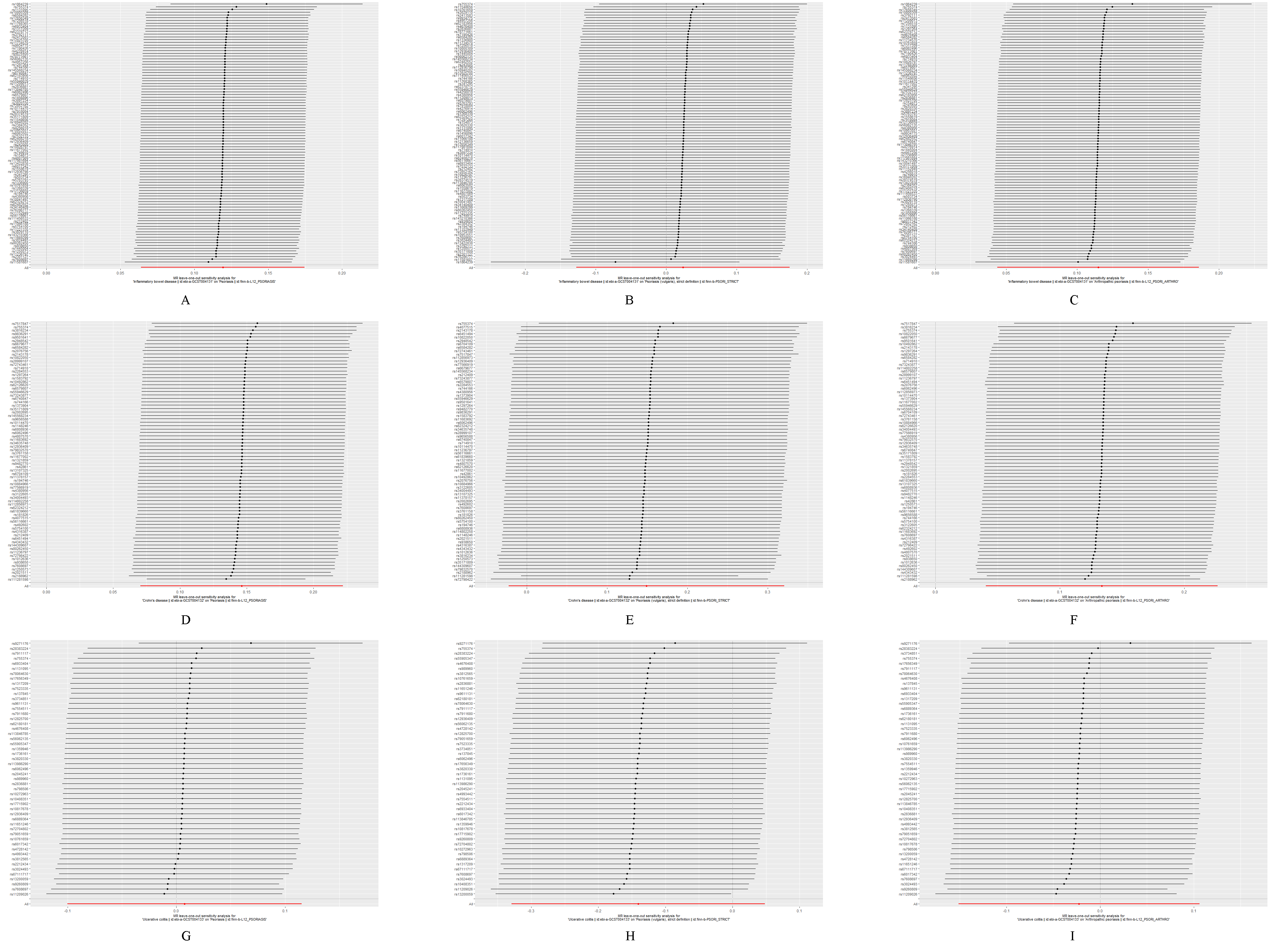


**Supplementary Figure 5.** Leave one out sensitivity tests of MR analyses of inflammatory bowel disease and its the subtypes on psoriasis as a whole, psoriasis vulgaris and psoriatic arthritis. Calculate the MR results of the remaining IVs after removing the IVs one by one. A: Primary outcome (Osteoarthritis); B: Secondary outcome (Knee Osteoarthritis); C: Secondary outcome (Hip Osteoarthritis). IBD, inflammatory bowel disease; CD: Crohn’s disease; UC: ulcerative colitis; PSO: psoriasis; PV: psoriasis vulgaris; PsA: psoriatic arthritis. A: IBD on PSO; B: IBD on PV; C: IBD on PsA; D: CD on PSO; E: CD on PV; F: CD on PsA; G: UC on PSO; H: UC on PV; I: UC on PsA.


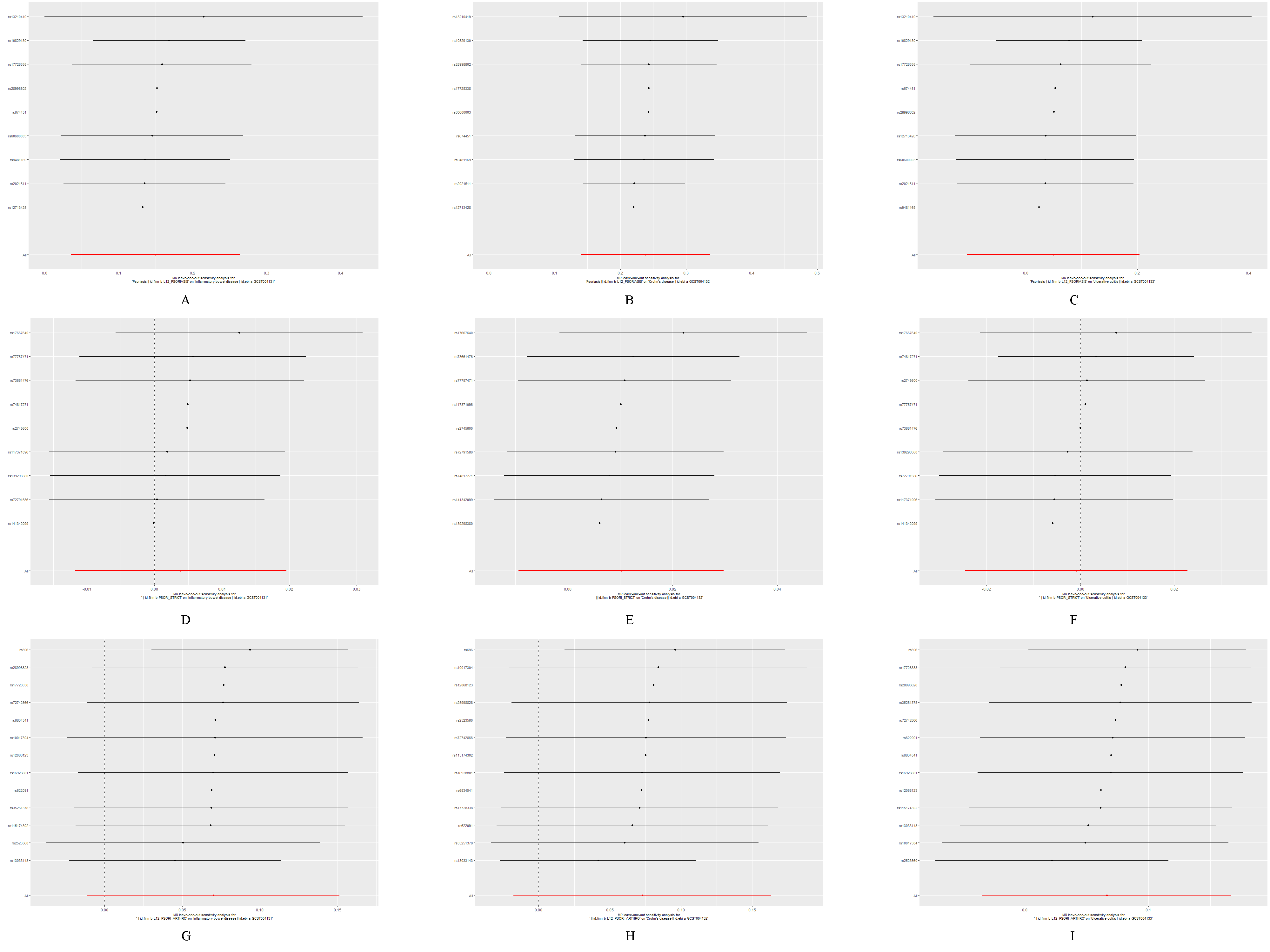


**Supplementary Figure 6.** Leave one out sensitivity tests of MR analyses of psoriasis and its subtypes on inflammatory bowel disease, Crohn’s disease and ulcerative colitis. Calculate the MR results of the remaining IVs after removing the IVs one by one. A: Primary outcome (Osteoarthritis); B: Secondary outcome (Knee Osteoarthritis); C: Secondary outcome (Hip Osteoarthritis). IBD, inflammatory bowel disease; CD: Crohn’s disease; UC: ulcerative colitis; PSO: psoriasis; PV: psoriasis vulgaris; PsA: psoriatic arthritis. A: PSO on IBD; B: PSO on CD; C: PSO on UC; D: PV on IBD; E: PV on CD; F: PV on UC; G: PsA on IBD; H: PsA on CD; I: PsA on UC.
